# Thyroid Malignancy among Fine Needle Aspiration Cytology of Thyroid Lesions in a Tertiary Care Centre

**DOI:** 10.31729/jnma.8396

**Published:** 2024-01-31

**Authors:** Suman Gurung, Niranjan Karki, Kanchan Bogati, Sunil Baniya, Swapnil Shah, Anupam Sharma Nepal, Rajya Khadka, Jyoti Lamichhane

**Affiliations:** 1 Department of Pathology, Shree Birendra Hospital, Chhauni, Kathmandu, Nepal; 2 Department of Pathology, Bhaktapur Hospital, Dudhpati, Bhaktapur, Nepal; 3 Patan Academy of Health Sciences, Lagankhel, Lalitpur, Nepal; 4 Nepalese Army Institute of Health Sciences, Sanobharyang, Kathmandu, Nepal; 5 Nepalgunj Medical College Teaching Hospital, Kohalpur, Nepalgunj, Nepal

**Keywords:** *cytology*, *malignancy*, *prevalence*, *thyroid*

## Abstract

**Introduction:**

The most prevalent endocrine cancer in the world is thyroid cancer, and its incidence is on the rise. The distinction between benign and malignant thyroid nodules must be made, which is why fine needle aspiration cytology of thyroid lesion is necessary and required. This study aimed to find out the prevalence of thyroid malignancy among fine needle aspiration cytology of thyroid lesions in a tertiary care centre.

**Methods:**

A descriptive cross-sectional study was conducted among fine needle aspiration cytology of thyroid lesions in a tertiary care centre after obtaining ethical approval from the Institutional Review Committee. Data from 13 April 2020 to 13 April 2023 was collected between 19 May 2023 to 19 June 2023. All the patients with complete hospital record data were included in this study. However, repetitive fine needle aspiration cytology of thyroid lesion were excluded from the study. Fine needle aspiration cytology diagnoses were categorized in this study as per the Bethesda system for reporting thyroid cytopathology. The point estimate was calculated at a 95% Confidence Interval.

**Results:**

Among 398 fine needle aspiration cytology of thyroid lesions, thyroid malignancy was seen in 12 (3.02%) (1.34-4.70, 95% Confidence Interval) patients.

**Conclusions:**

The prevalence of thyroid malignancy was found to be lower than other studies done in similar settings.

## INTRODUCTION

Fine needle aspiration cytology (FNAC) is recognized as a cost-effective, minimally invasive, low-complication, non-operative diagnostic procedure for the majority of thyroid lesions.^1^ It is crucial to distinguish between benign and malignant thyroid nodules, as surgery is required for malignant nodules and FNAC is considered the gold standard for diagnosing them.^2^

The prevalence of thyroid cancer is increasing globally, and it is the most prevalent endocrine cancer worldwide.^3^ About 1-5% of all cancers in women and less than two percent in men worldwide are thyroid cancers.^4^ About 5-10% of thyroid lesions are malignant and require surgical and pharmaceutical treatment.^5^

Numerous studies have been conducted on thyroid disorders, however, the prevalence of thyroid malignancy diagnosed through FNAC has been less studied.

This study aimed to find out the prevalence of thyroid malignancy among fine needle aspiration cytology of thyroid lesion in a tertiary care centre.

## METHODS

This descriptive cross-sectional study was conducted among the FNACs in the Department of Pathology of the Shree Birendra Hospital, Chhauni, Kathmandu, Nepal. Data from 13 April 2020 to 13 April 2023 were collected between 19 May 2023 to 19 June 2023 from the hospital records. The ethical approval was taken from the Institutional Review Committee of the Nepalese Army Institute of Health Sciences (Reference number: 763). All the patients sent to the Department of Pathology for FNACs with complete hospital record data and categorized according to the Bethesda System for Reporting Thyroid Cytopathology (TBSRTC) were included in this study. Patients with repetitive FNACs were excluded from the study. Convenience sampling method was used. The sample size was calculated using the following formula:


$$\begin{array}{ll} \text{n} & ={\text{Z}}^{2}\times \frac{\text{p}\times \text{q}}{{\text{e}}^{\text{2}}}\\ & =1.96^{2}\times \frac{0.50\times 0.50}{0.05^{2}}\\ & =385 \end{array}$$

Where,

n = minimum required sample size
Z = 1.96 at 95 % Confidence Interval
p = prevalence taken as 50% for maximum sample size
q = 1-p
e = margin of error, 5%

The minimum required sample size was 385. However, the final sample size taken was 398.

FNAC record books were assessed to extract patient demographic data, clinical characteristics, and FNAC diagnosis from the pathology department. FNAC diagnoses were categorized in this study as per the Bethesda system for reporting thyroid cytopathology.^6^

Data were entered and analyzed using Microsoft Excel 2019. The point estimate was calculated at a 95% CI.

## RESULTS

Among 398 FNACs, the prevalence of thyroid malignancy was found to be 12 (3.02%) (1.34-4.70, 95% CI). Papillary thyroid carcinoma (PTC) accounted for the majority of cases 9 (75%), followed by poorly differentiated thyroid carcinoma (PDTC) 2 (16.67%) (Table 1).

**Table 1:** Diagnosis of thyroid malignancy (n= 12).

Diagnosis	n (%)
Papillary thyroid carcinoma	9 (75)
Poorly differentiated thyroid carcinoma	2 (16.67)
Medullary thyroid carcinoma	1 (8.33)

The majority of malignant cases were seen among females 10 (83.33%) (Table 2).

**Table 2:** Gender-wise distribution of patients with thyroid malignancy (n= 12).

Gender	n (%)
Female	10 (83.33)
Male	2 (16.67)

## DISCUSSION

In this study, the prevalence of thyroid malignancy was found to be 12 (3.02%). This rate demonstrating a relatively lower incidence of thyroid malignancy, stands in contrast to findings from similar studies conducted in various geographical regions. Notably, when compared to studies in Pakistan, the prevalence in the present study was lower, with the reported rate in Pakistan being 11%. Likewise, the observed prevalence in Saudi Arabia (6.1%), Ethiopia (11.5%), and the United Kingdom (18.3%) surpassed the findings of the current investigation.^3,4^ This could be due to differences in variances in the definition of malignancy, the selection of study participants, the methods employed for data collection and analysis, and differences in the sample size can contribute to the observed differences.

Consistent with findings from comparable studies, PTC emerges as the predominant category of diagnosed malignancy, constituting 75% of the cases in our study. This aligns with the investigations conducted in India, where PTC accounts for a prevalence range of 60-70%, and in Nepal, where it represents a substantial 77.42%.^7,9^ PTC may be more prevalent due to its relation with radiation exposure and high iodine consumption.^10^ In alignment with the predominant findings across various studies, our research also concurs that thyroid cancers predominantly affect the female population. This observation underscores the consistency in the demographic profile of individuals most commonly affected by thyroid malignancy, emphasizing the significance of female gender as a notable factor in the prevalence of this disease.^9,11^ It has been said that the gender disparity in thyroid malignancy is due to the fluctuation of sex hormones during a woman's menstrual cycle and pregnancy.^12^

This study has a few limitations. Since this is a descriptive cross-sectional study conducted at a single institution, the results cannot be generalized to the entire population. In addition, a study design with a higher evidence level is recommended for future research.

## CONCLUSIONS

The prevalence of thyroid malignancy in our study was found to be lower compared to other studies done in a similiar settings. This underscores the importance of investigating etiological factors and considering potential implications for diagnostic and therapeutic strategies. Additionally, it is recommended to conduct an analytical study with a focus on the association between gender and thyroid malignancy for a more comprehensive understanding.
